# Homogenization and Localization of Ratcheting Behavior of Composite Materials and Structures with the Thermal Residual Stress Effect

**DOI:** 10.3390/ma12183048

**Published:** 2019-09-19

**Authors:** Danhui Yang, Zhibo Yang, Zhi Zhai, Xuefeng Chen

**Affiliations:** 1 The State Key Laboratory for Manufacturing Systems Engineering, Xi’an 710054, China; 2 School of Mechanical Engineering, Xi’an Jiaotong University, Xi’an 710049, China

**Keywords:** metal matrix composites, finite-volume theory, homogenization, cyclic plasticity, ratcheting effect, residual stresses

## Abstract

In this contribution, the ratcheting behavior and local field distribution of unidirectional metal matrix composites are investigated under cyclic loading. To that end, we extended the finite-volume direct averaging micromechanics (FVDAM) theory by incorporating the rule of nonlinear kinematic hardening. The proposed method enables efficient and accurate simulation of the ratcheting behavior of unidirectional composites. The local satisfaction of equilibrium equations of the FVDAM theory facilitates quick and rapid convergence during the plastic iterations. To verify the proposed theory, a finite-element (FE) based unit cell model is constructed with the same mesh discretization. The remarkable correlation of the transverse response and local field distribution generated by the FVDAM and FE techniques demonstrates the effectiveness and accuracy of the proposed models. The stress discontinuities along the fiber/matrix interface that are generic to the finite-element theory are absent in the FVDAM prediction. The effects of thermal residual stresses induced during the consolidation process, as well as fiber orientations, are revealed. The generated results indicate that the FVDAM is well suited for simulating the elastic-plastic ratcheting behavior of metal matrix composites, which will provide the conventional finite-element based technique with an attractive alternative.

## 1. Introduction

Metal matrix composites have become and will continue to be one of the most attractive alternatives to conventional materials for many applications in engineering such as turbine engine components, pressure pipelines and vessels. The applications often leave the composites exposed to complicated cyclic loading, which leads to cyclic deformations including ratcheting and endangers safety in the operation. For the safety and full use of the metal matrix composites, a constitutive relation needs to be established to characterize the ratcheting effect of the metal matrix composites. The properties of composites are usually decided by constituent material properties and microstructure parameters. These factors make it very difficult to characterize the mechanical behavior. In addition, during the fabrication of the composites, materials are usually consolidated at a high temperature which induces the residual stresses when being cooled to room temperature. The residual stresses may change the material properties and have a great influence on the thermo-mechanical response. Therefore, the study of the thermal residual effect is critical to the accurate prediction of the ratcheting effect in the metal matrix composites.

Ratcheting is a cyclic accumulation of inelastic deformation, which occurs to materials during cyclic loading with no-zero mean stresses. It has been a critical factor in fatigue life prediction and structure designs. However, simulating ratcheting is a complicated task since the inelastic deformation increases with loading cycle. The ratcheting of monotonic homogeneous metal materials is investigated extensively; one of the most famous cyclic plasticity models is the Armstrong and Frederick model (AF model) [1]. The terms of dynamic recovery of the AF model enable the calculation of the ratcheting effect. However, the dynamic recovery terms in this model are too active, which leads to overprediction of the ratcheting effect. Many researchers have extended the AF model to improve the precision of the prediction, such as Ohno and Wang [2,3,4], Tanaka [5], Chaboche [6,7,8], Abdel-Karim and Ohno [9] and Kang [10]. Details of these works can be seen in the review papers by Ohno [11], Bari and Hassan [12], Chaboche [8], Kang [13], Sajjad [14] and Chen [15]. Meanwhile, experimental characterizations of the cyclic deformations of metal matrix composites have been conducted by researchers, cf., Zhang et al. [16], Kang et al. [17], Han et al. [18] and Kimmig et al. [19]. Compared with monotonic homogeneous metal materials, the properties of the metal matrix composites are decided by off-axis angles, volume fractions and array patterns. Therefore, they must be tested repeatedly for each variation of the composites, which makes the experimental characterization of composite materials a more tedious and expensive task.

Numerical modeling of ratcheting of the composites has been investigated by many researchers during the past decades [20,21,22,23,24,25]. The classical micromechanics models under the framework of the Eshelby theory [26] and the Mori–Tanaka model [20,21] were built to simulate the mechanical response of metal matrix composites. However, classical micromechanics is based on the total deformation theory and cannot simulate the ratcheting effect of composite materials.Therefore, some researchers have built the model based on tangent incremental linearization theory [27] to simulate the elastoplastic response and additive scheme [28] or variational approaches [29] for the viscoplastic response of composites. Conventionally, the finite-element method (FEM) is the most commonly used method to simulate the elastoplastic response of the composites. However, care must be taken since the large modulus contrast near the fiber and matrix interface can produce stress concentrations which requires a more detailed mesh for FEM to avoid stress discontinuity at the interface.

The finite-volume direct averaging micromechanics (FVDAM) theory [30] has become an appealing alternative to the conventional FEM method for simulating composite materials. Many researchers have developed the theory extensively over the years [31,32,33,34]. The viscoelasticity problem [35,36,37], damage model [38], the effect of surface energy [39,40], finite deformation [41,42] and multiphysics behavior [34,43,44,45] are incorporated with FVDAM.

While the FVDAM theory has been studied extensively, little work has been reported concerning the cyclic plasticity. The semi-analytical framework, quick convergence and excellent stability of the FVDAM theory make it particularly suitable for analyzing the cyclic behavior of metal matrix composites.

Therefore, in this research, the FVDAM is further extended to incorporate the ratcheting plasticity model, which allows efficient and accurate simulations of the thermo-mechanical cyclic behavior of the metal matrix composites. The proposed model not only enables the prediction of global ratcheting effects but also provides local information of the microstructures of the metal matrix composites. The validation of the model is provided by FEM simulations. The influence of thermal residual stresses, as well as off-axis angles, is discussed.

The main contributions of this research are listed as follows:

(1) The cyclic plastic constitutive model is implemented successfully into the FVDAM theory for the first time, giving the FVDAM the ability to simulate the cyclic behavior of the metal matrix composites.
(2) Not only the homogenized cyclic response of the composites but also the local field distributions are obtained using the proposed model, since most of the mean-field and nongeometrical models cannot obtain the local field response.
(3) The effects of thermal residual stresses induced during the consolidation process, as well as fiber orientations, are considered, which makes the calculation of the cyclic behavior closer to the actual working condition.
(4) The effectiveness of the new micromechanical model to accurately simulate both the homogenized stress–strain curve and local field distribution is verified by an FEM model with the same mesh discretizations, providing a golden standard for the simulation. The results also show that the proposed model has a better convergence near the stress concentration region.

This paper is presented as follows: Section 2 gives an introduction of the framework of the parametric finite volume direct averaging micromechanics (FVDAM). The process of implementing the Abdel–Karim–Ohno constitutive model into parametric FVDAM theory is demonstrated in Section 3. Section 4 shows the homogenized and local response simulated by the proposed model and provides the validation with FEM theory.

## 2. Finite-Volume Direct Averaging Micromechanics (FVDAM)

**Figure 1:**
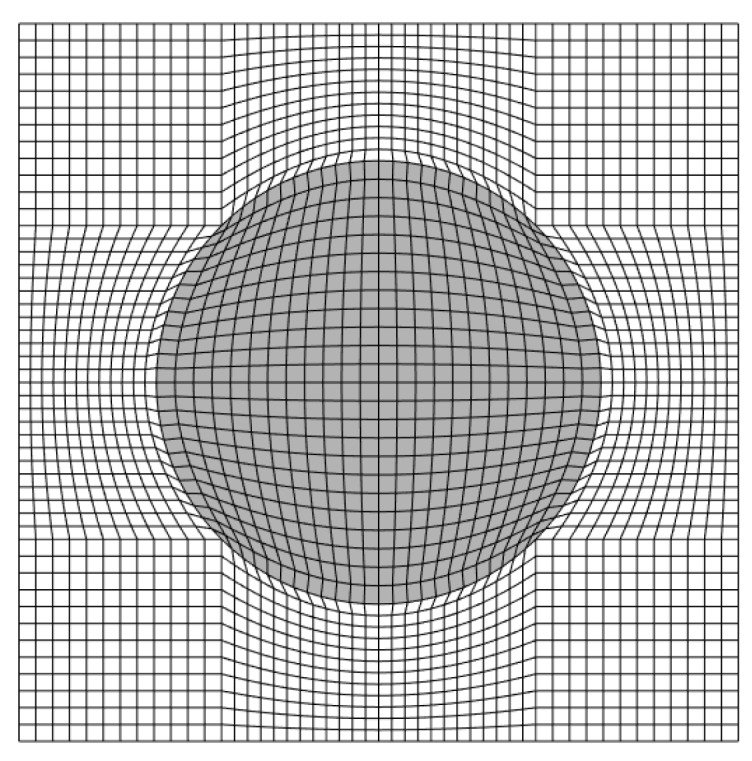
Unit cell discretizations.

The displacement equations of the parametric FVDAM are presented by a multiscale expansion. The index of the quadrilateral subvolumes are noted as (q). In this case, the study of unidirectional composites can be seen as a generalized plane strain problem, where fiber direction $y_{1}$ cannot affect stress components in other directions. Therefore, a two-dimensional unit cell can be used to analyze this problem (see Figure 1). The number of vertices $\left(y_{2}^{\left(m,q\right)},y_{3}^{\left(m,q\right)}\right)$ and the numbers of the faces $F_{m}$ are numbered counterclockwise. The detailed expressions of unit normal vector $n^{\left(m,q\right)}=\left[n_{2}^{\left(m,q\right)}n_{3}^{\left(m,q\right)}\right]$ for the face $F_{m}$ are:

(1) $$n_{2}^{\left(m,q\right)}=\frac{y_{3}^{\left(m+1,q\right)}-y_{3}^{\left(m,q\right)}}{L_{m}},n_{3}^{\left(m,q\right)}=\frac{y_{2}^{\left(m+1,q\right)}-y_{2}^{\left(m,q\right)}}{L_{m}}$$

where $L_{m}=\sqrt{{\left(y_{2}^{\left(m+1,q\right)}-y_{2}^{\left(m,q\right)}\right)}^{2}+{\left(y_{3}^{\left(m+1,q\right)}-y_{3}^{\left(m,q\right)}\right)}^{2}}$.

**Figure 2:**
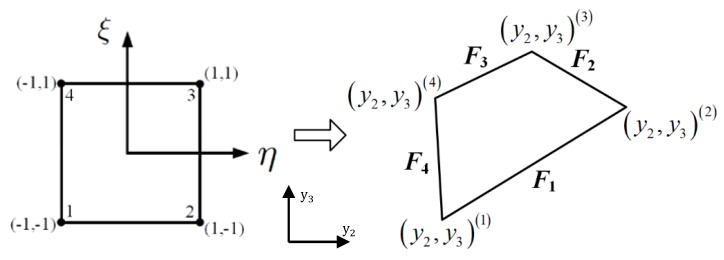
Parametric mapping.

The qth quadrilateral subvolume is generated by the transformation from the reference coordinates η−ξ to the actual discretized microstructure (see Figure 2):

(2) $$y_{i}^{\left(q\right)}\left(\eta ,\xi \right)=\sum_{m=1}^{4}N_{m}\left(\eta ,\xi \right)y_{i}^{\left(m,q\right)},i=2,3$$

where

(3) $$\begin{array}{c} N_{1}\left(\eta ,\xi \right)=\left(1/4\right)\left(1-\eta \right)\left(1-\xi \right),N_{2}\left(\eta ,\xi \right)=\left(1/4\right)\left(1+\eta \right)\left(1-\xi \right)\\ N_{3}\left(\eta ,\xi \right)=\left(1/4\right)\left(1+\eta \right)\left(1+\xi \right),N_{4}\left(\eta ,\xi \right)=\left(1/4\right)\left(1-\eta \right)\left(1+\xi \right) \end{array}$$

The displacement equation in each qth subvolume is generated by using the two-scale expansion in the slow scale **x** and fast scale **y**:

(4) $$u_{i}^{\left(q\right)}\left(x,y\right)={\bar{\varepsilon }}_{ij}x_{j}+u_{i}^{\prime (q)}\left(y\right)$$

which produces the local strain–displacement relations in the actual coordinates $y_{2}-y_{3}$ with the form:

(5) $$\varepsilon_{ij}^{\left(q\right)}={\bar{\varepsilon }}_{ij}+\varepsilon_{ij}^{\prime (q)}={\bar{\varepsilon }}_{ij}+\frac{1}{2}\left(\frac{\partial u_{i}^{\prime (q)}}{\partial y_{j}}+\frac{\partial u_{j}^{\prime (q)}}{\partial y_{i}}\right)$$

where $\varepsilon_{ij}^{\prime (q)}$ are the fluctuating strain components. The second order Legendre expansion is applied to approximate the $u_{i}^{\prime (q)}\left(i=1,2,3\right)$:

(6) $$u_{i}^{\prime (q)}=W_{i(00)}^{\left(q\right)}+\eta W_{i(10)}^{\left(q\right)}+\xi W_{i(01)}^{\left(q\right)}+\frac{1}{2}\left(3\eta^{2}-1\right)W_{i(20)}^{\left(q\right)}+\frac{1}{2}\left(3\xi^{2}-1\right)W_{i(02)}^{\left(q\right)}$$

where $W_{i(mn)}^{\left(q\right)}$ are unknown coefficients which can be expressed by displacement and tractions in the *q*th subvolume.

The interfacial displacements and tractions on the pth face are given below:

For face (p=1,3):

$${\hat{u}}_{i}^{\left(p,q\right)}=\frac{1}{2}\int_{-1}^{1}u_{i}^{\prime (q)}\left(\eta ,\mp 1\right)d\eta ,\;{\hat{t}}_{i}^{\left(p,q\right)}=\frac{1}{2}\int_{-1}^{1}t_{i}^{\left(q\right)}\left(\eta ,\mp 1\right)d\eta$$

For face (p=2,4):

(7) $${\hat{u}}_{i}^{\left(p,q\right)}=\frac{1}{2}\int_{-1}^{1}u_{i}^{\prime (q)}\left(\pm 1,\xi \right)d\xi ,\;{\hat{t}}_{i}^{\left(p,q\right)}=\frac{1}{2}\int_{-1}^{1}t_{i}^{\left(q\right)}\left(\pm 1,\xi \right)d\xi$$

where $t_{i}^{\left(p,q\right)}=\sigma_{ij}^{\left(p,q\right)}n_{j}^{\left(p,q\right)}$ and $\sigma_{ij}^{\left(p,q\right)}=C_{ijkl}^{\left(q\right)}{\left(\varepsilon_{kl}-\varepsilon_{kl}^{pl}-\varepsilon_{kl}^{th}\right)}^{\left(p,q\right)}$, from Hook’s law.

In the FVDAM theory, the inverse of the volume-averaged Jacobian $\hat{J}$ is applied to mapping the surface-averaged interfacial displacement in the actual coordinates $y_{2}-y_{3}$ onto the reference coordinates η−ξ. The surface-averaged interfacial displacement in η−ξ can be expressed by the first and second order coefficients in representation of the displacement field; these coefficients can be written as $W_{i}={\left[W_{i(10),}W_{i(01),}W_{i(20),}W_{i(02)}\right]}_{}^{T}$. Therefore, we have:

(8) $${\left[\begin{array}{c} \frac{\hat{\partial {u^{\prime }}_{i}}}{\partial y_{2}}\\ \frac{\hat{\partial {u^{\prime }}_{i}}}{\partial y_{3}} \end{array}\right]}^{\left(i\right)}=\hat{J}{\left[\begin{array}{c} \frac{\hat{\partial {u^{\prime }}_{i}}}{\partial \eta }\\ \frac{\hat{\partial {u^{\prime }}_{i}}}{\partial \xi } \end{array}\right]}^{\left(\hat{i}\right)}=\hat{J}A_{i}W_{i}$$

where

(9) $$A_{1,3}=\left[\begin{array}{cccc} 1 & 0 & 0 & 0\\ 0 & 1 & 0 & \pm 3 \end{array}\right],\;A_{2,4}=\left[\begin{array}{cccc} 1 & 0 & \pm 3 & 0\\ 0 & 1 & 0 & 0 \end{array}\right]$$

By using the surface-averaged subvolume equilibrium equations:

(10) $$\sum_{p=1}^{4}\int {}_{L_{p}}\sigma^{\left(p,q\right)}\times \;n^{\left(p,q\right)}dl=\sum_{p=1}^{4}\int {}_{L_{p}}t^{\left(p,q\right)}dl=\sum_{p=1}^{4}L_{p}{\hat{t}}^{\left(p,q\right)}=0$$

where $L_{p}$ represents the length of the pth face, the expressions of $W_{i}$ in the qth subvolume can be expressed by ${\hat{u}}^{\prime (q)}$.

By using the relation between traction and fluctuating displacements in a surface-average sense, the following equation can be derived:

(11) $${\hat{T}}^{\left(q\right)}=K^{\left(q\right)}{\hat{U}}^{\prime (q)}+N^{\left(q\right)}C^{\left(q\right)}\bar{\varepsilon }+\Gamma^{\left(q\right)}+G^{\left(q\right)}$$

where ${\hat{T}}^{\left(q\right)}={\left[{\hat{t}}^{\left(1\right)}{\hat{t}}^{\left(2\right)}{\hat{t}}^{\left(3\right)}{\hat{t}}^{\left(4\right)}\right]}^{(q)T}$, ${\hat{U}}^{\prime (q)}={\left[{\hat{u}}^{\prime (1)}{\hat{u}}^{\prime (2)}{\hat{u}}^{\prime (3)}{\hat{u}}^{\prime (4)}\right]}^{(q)T}$, $N^{\left(q\right)}$ denotes the unit vectors that show the orientation of each of the four faces of the qth subvolume, and $\Gamma^{\left(q\right)}$ and $G^{\left(q\right)}$ contain vectors concerning thermal and plastic contributions. Each element in ${\hat{T}}^{\left(q\right)}$ and ${\hat{U}}^{\prime (q)}$ has three components, ${\hat{t}}^{\left(p,q\right)}={\left[{\hat{t}}_{1}{\hat{t}}_{2}{\hat{t}}_{3}\right]}^{\left(p,q\right)}$ and ${\hat{u}}^{\prime (p,q)}={\left[{{\hat{u}}^{\prime }}_{1}{{\hat{u}}^{\prime }}_{2}{{\hat{u}}^{\prime }}_{3}\right]}^{\left(p,q\right)}$. The local stiffness matrix $K^{\left(q\right)}$ contains 16 submatrices $K_{ij}^{\left(q\right)}$ and the submatrix $K_{ij}^{\left(q\right)}$ is a 3×3 matrix derived by the geometric and mechanical properties in each subvolume.

The global stiffness matrix can then be assembled using the continuity equations:

(12) $$\begin{array}{c} {\hat{u}}^{\prime (2,q-1)}={\hat{u}}^{\prime (4,q)},{\hat{u}}^{\prime (3,\bar{q}-1)}={\hat{u}}^{\prime (1,\bar{q})}\\ {\hat{t}}^{\left(2,q-1\right)}+{\hat{t}}^{\left(4,q\right)}=0,{\hat{t}}^{\left(3,\bar{q}-1\right)}+{\hat{t}}^{\left(1,\bar{q}\right)}=0 \end{array}$$

Then the global system of equations can be formed after applying periodic boundary conditions and eliminating the singularity of the global stiffness matrices. The resulting systems are expressed below:

(13) $$K^{global}{\hat{U}}^{\prime }=\Delta C\bar{\varepsilon }+\Gamma +G$$

where ΔC contains the differences of adjacent local stiffness matrices, Γ and G represent the thermal effect and plastic information. The fluctuating surface-averaged interfacial displacements ${\hat{U}}^{\prime }$ can be determined by solving Equation (13). The coefficients $W_{i}$ can be calculated subsequently, which enables the calculation of local fields in every subvolume.

The results of ${\hat{U}}^{\prime }$ can then be used to derive the Hill’s strain concentration matrix $A^{\left(q\right)}$ by using the relation below:

(14) $${\bar{\varepsilon }}^{\left(q\right)}=A^{\left(q\right)}\bar{\varepsilon }+D^{\left(q\right)}$$

where the components of $A^{\left(q\right)}$ are derived by applying only one component of macroscopic strain at a time. $D^{\left(q\right)}$ is a vector concerning thermal and plastic contributions, which can be presented by macroscopic strains $\bar{\varepsilon }$ at each loading step.

The homogenized Hook’s law can be calculated by taking the volume average of the stress field as below:

(15) $$\bar{\sigma }=\frac{1}{V}\int \sigma (x)dV=\frac{1}{V}\sum_{q=1}^{N_{q}}\int_{V_{q}}{\bar{\sigma }}^{\left(q\right)}\left(x\right)dV^{\left(q\right)}=\sum_{q=1}^{N_{q}}v^{\left(q\right)}{\bar{\sigma }}^{\left(q\right)}$$

where $v^{\left(q\right)}=V^{\left(q\right)}/V$ represents the volume fraction.

The macroscopic constitutive equations for the composite material can be derived using the localization relations and stress–strain relations in the volume-averaged sense

(16) $$\bar{\sigma }=C^{*}\bar{\varepsilon }-\left({\bar{\sigma }}^{th}+{\bar{\sigma }}^{pl}\right)$$

where $C^{*}=\sum_{q=1}^{N_{q}}v^{\left(q\right)}C^{\left(q\right)}A^{\left(q\right)}$ represents the homogenized stiffness matrix; the volume-averaged thermal and plastic stresses can be derived below

(17) $${\bar{\sigma }}^{th}+{\bar{\sigma }}^{pl}=-\sum_{q=1}^{N_{q}}v^{\left(q\right)}\left[C^{\left(q\right)}D^{\left(q\right)}-\Gamma^{\left(q\right)}\Delta T-{\bar{\sigma }}^{pl(q)}\right]$$

The vector G in Equation (13) denotes plastic contributions, which are an integration of plastic strains on the surface of each subvolume. The total plastic strain at each material point consists of the contribution of the previous step and the contribution of current load increment

(18) $$\varepsilon_{}^{pl(q)}\left(\eta ,\xi \right)=\varepsilon_{}^{pl(q)}\left(\eta ,\xi \right)\left|{}_{previous}+\right.d\varepsilon_{}^{pl(q)}\left(\eta ,\xi \right)$$

In order to give the FVDAM model the capability of simulating the ratcheting effect, a cyclic plastic constitutive relation should be implemented in this model when calculating the plastic strain increment $d\varepsilon_{}^{pl(q)}\left(\eta ,\xi \right)$ in Equation (18). The detail of the implementation is described in Section 3.

## 3. Implementation of the Cyclic Plasticity Model

After the discretization in Section 2, the aim of the model becomes the calculation of the plastic strain incremental of each and every material point in Equation (18). In this paper, we introduce the Abdel–Karim–Ohno model [9] into the FVDAM theory, since the Abdel–Karim–Ohno model is a low-order nonlinear constitutive model which makes it easy to implement in the algorithm [46]. We begin with a brief summary of the Abdel–Karim–Ohno model. For the elastoplastic problems, the small strain ε is assumed to be a combination of elastic deformation $\varepsilon^{e}$ and plastic deformation $\varepsilon^{pl}$ and back stresses α are divided into *N* parts as shown below:

(19) $$\varepsilon =\varepsilon^{e}+\varepsilon^{pl}$$

(20) $$\alpha =\sum_{i=1}^{N}\alpha_{i}$$

Then, by using Hook’s law and the flow rule related to Mises-type yield surface F=0, the function of $\varepsilon^{e}$ and $\varepsilon^{pl}$ in Equation (19) can be written as follows:

(21) $$\sigma =C:\varepsilon^{e}$$

(22) $${\dot{\varepsilon }}^{pl}=\dot{\lambda }\frac{\partial F}{\partial \sigma }$$

(23) $$F=\sqrt{\frac{3}{2}\left(s-a\right):\left(s-a\right)}-Y$$

where (:) means inner product calculation, C is the stiffness matrix, $\dot{\lambda }$ can be calculated using $\dot{F}=0$ and F=0, s and a are deviatoric parts of σ and α. Therefore, in the deviatoric space, a is the center of the yield surface and *Y* represents the radius of the yield surface.

Normally, a and *Y* will move and expand during the inelastic deformation. In this paper, we consider kinematic hardening, where the radius of the yield surface *Y* is constant and the center of the yield surface a moves. Since a is the deviatoric part of α, a can also be decomposed into *N* different parts:

(24) $$a=\sum_{i=1}^{N}a_{i}$$

The evolution of a is described as below:

(25) $${\dot{a}}_{i}=\zeta_{i}\left[\frac{2}{3}r_{i}{\dot{\varepsilon }}^{pl}-\mu_{i}a_{i}\dot{p}-H\left(f_{i}\right)\langle {\dot{\lambda }}_{i}\rangle a_{i}\right]$$

(26) $${\dot{\lambda }}_{i}={\dot{\varepsilon }}^{p}:\frac{a_{i}}{r_{i}}-\mu_{i}\dot{p}$$

where $\zeta_{i}$ is a material parameter, $\mu_{i}$ is the weighing coefficient ranging from 0 to 1, $\mu_{i}\;=\;1$ corresponds to the AF model, $\mu_{i}\;=\;0$ corresponds to the first version of the Ohno–Wang model, $r_{i}$ can be determined by the unidirectional tensile test and   denotes Macaulay’s brackets (i.e., $\langle x\rangle =x$ if x≥0, $\langle x\rangle =0$ if x<0). $f_{i}=3/2\left(a_{i}:a_{i}\right)-{r_{i}}^{2}$ represents a critical surface. *p* stands for the accumulated plastic strain, where:

(27) $$\dot{p}=\sqrt{\frac{2}{3}{\dot{\varepsilon }}^{pl}:{\dot{\varepsilon }}^{pl}}$$

For numerical implementation, the constitutive equations (Equations (19)–(25)) should be discretized over time *t*. The problem can then be described as follows: Knowing the variables of every point in the qth subvolume in the reference coordinates (i.e., $\sigma^{\left(q\right)}\left(\eta ,\xi \right)$, ${a_{i}}^{\left(q\right)}\left(\eta ,\xi \right)$, $\varepsilon^{\left(q\right)}\left(\eta ,\xi \right)$, $\varepsilon {}_{}^{pl(q)}\left(\eta ,\xi \right)$, $p^{\left(q\right)}\left(\eta ,\xi \right)$, $Y^{\left(q\right)}\left(\eta ,\xi \right)$) at $t_{n}$ and giving the strain increment $d\varepsilon^{\left(q\right)}\left(\eta ,\xi \right)$, try to find the plastic strain increment $d\varepsilon {}_{}^{pl(q)}\left(\eta ,\xi \right)$ at $t_{n+1}$, which can be solved by applying the backward Euler method and the return mapping method [46].

After the plastic strain increment $d\varepsilon {}_{}^{pl(q)}\left(\eta ,\xi \right)$ is calculated, Equation (18) can be solved and vector G in Equation (13) can be derived, which allows the updating of $D^{\left(q\right)}$ in Equation (14) and the follow-up calculation of the homogenized and local response of the composites under cyclic loading.

**Figure 3:**
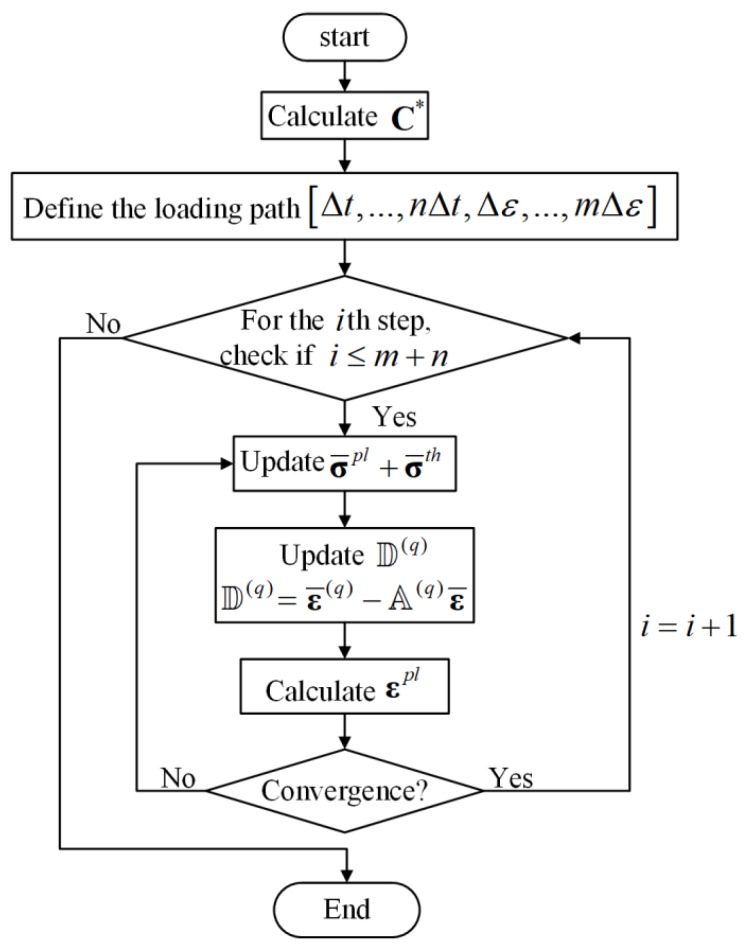
Flow chart.

The whole algorithm is illustrated in Figure 3. The outer loop controls the macroscopic loading path. We start with a thermal cooling process (ΔT=−550 ${}^{\circ }C$), which has been discreted into *n* parts [Δt,…,nΔt=ΔT]. Then, we apply the macroscopic mechanical loading, which has been discreted into *m* parts [Δε,…,mΔε].

The inner loop demonstrates the process of obtaining the local and homogenized response of the composite material under thermal and cyclic mechanical loading. The effective thermal expansion constants are used to generate thermal residual stresses ${\bar{\sigma }}^{th}$ of the composites to simulate the cooling process. After thermal loading, cyclic mechanical loading is applied and the cyclic plasticity constitutive equation is implemented to calculate cyclic plastic strain $\varepsilon {}_{}^{pl}$ in the *q*th subvolume. When the plastic strain in all subvolumes meets the requirement of convergence, the local response can be obtained. Then the homogenized response can be updated using the volume-averaging method.

During the calculation of the plastic strain in the qth subvolume, we applied the Newton–Raphson method, the convergence of which is quadratic [47]. The convergence criterion is written in terms of the accumulated plastic strain increment $\Delta p_{i+1}^{\left(q\right)}$ as follows:

(28) $$\left|1-\Delta p_{i+1}^{\left(q\right)}\left(k-1\right)/\Delta p_{i+1}^{\left(q\right)}\left(k\right)\right|<10^{-4},k=1,2,3,\ldots$$

where *k* denotes the number of local iterations and *i* represents the loading step.

The numerical successive method we use is similar to the one proposed by Kaboyashi and Ohno [46]. The detailed demonstration of convergence can also be found in [46].

## 4. Validation and Numerical Results

### 4.1. Unidirectional Transverse Loading

In order to verify the effectiveness of the FVDAM theory to accurately predict both the homogenized stress–strain curve and local field distribution, a finite-element unit cell model is built with the same mesh discretizations, providing a golden standard for the FVDAM simulation.

In this paper, we simulated the strain-controlled unidirecional loading experiment, which means we control the macroscopic ${\bar{\varepsilon }}_{22}$. However, other components of $\bar{}\}\varepsilon$ are not zero. As in the unidirectional experiment, we only load on direction 2 and other directions are not constrained on the material. Therefore, knowing ${\bar{\varepsilon }}_{22}$ and the global stiffness matrix, other components of $\bar{\varepsilon }$ can be calculated using Hook’s law.

**Figure 4:**
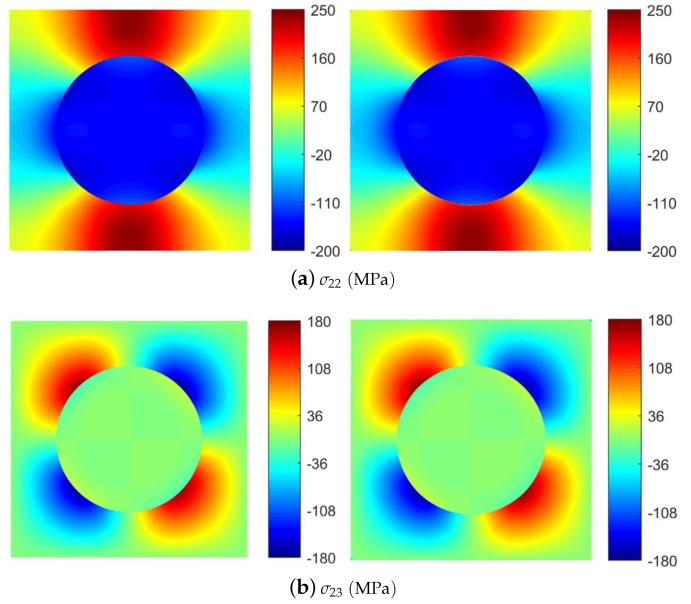
Comparison of selected local stress distributions after thermal cooldown temperature of $\Delta T=-550{\;}^{\circ }C$: FEM (left); FVDAM (right).

To simulate the response of the composite materials with thermal residual stresses, a square unit cell with a fiber was built. The volume fraction of fiber is 0.3. The material properties are listed in Table 1 and Table 2. The large modulus contrast and the strong fiber–fiber interaction at this volume fraction produce highly localized stress fields, which are very demanding for the accuracy of the method. We first compare the local stress distributions within the composite microstructure after a cool down temperature of ΔT=−550 ${}^{\circ }C$ simulated by the FVDAM and FEM analyses (see Figure 4). The thermal expansion constants $\alpha^{*}=C^{*-1}\sum_{q=1}^{N_{q}}{\nu^{\left(q\right)}\left(A^{\left(q\right)}\right)}^{T}\Gamma^{\left(q\right)}$ are obtained using the results of Levin [48]. Because the composites are not constrained during the consolidation process, the macroscopic stresses in Equation (16) are taken as zeros. To generate the smooth thermal stress fields, the unit cells were discretized into 96×96 subvolumes/elements in both approaches. We note that the thermal residual stresses of the present material systems generated by both approaches are in good agreement, illustrating the excellent predictive capability of the FVDAM theory.

**Figure 5:**
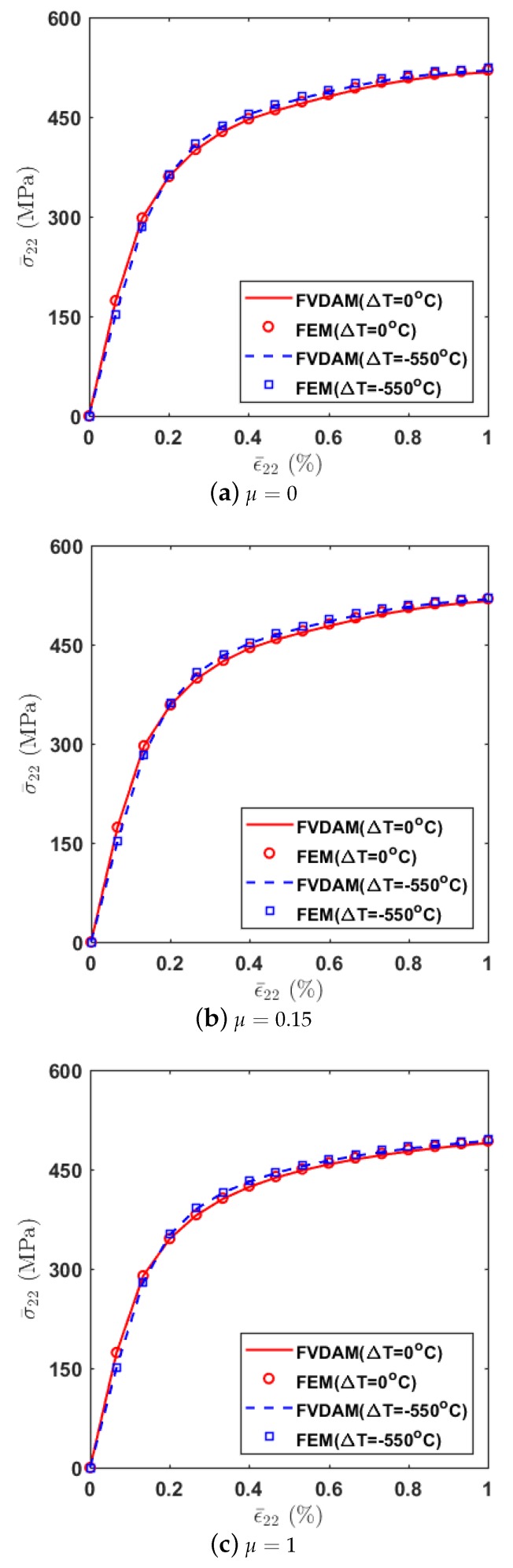
Comparison of the transverse thermo-mechanical tensile response with different μ.

To illustrate the effect of thermal residual stresses, Figure 5 compares the transverse thermo-mechanical tensile response of the composite with different values of μ=0,0.15,1. The temperature changes are prescribed as ΔT=−550 ${}^{\circ }C$. The stress–strain response generated without thermal residual stress effects ($\Delta T=0{\;}^{\circ }C$) are included in the figures for comparison. For verification purposes, the corresponding FEM results under the same loading conditions are enclosed in the figures as well. As observed, the correlations between the two methods are in good agreement. The thermal residual stresses provide a slight enhancement in homogenized stresses in the elastic-plastic region while the elastic response almost remains the same. A close look at the figures also indicates that the initiation of plasticity in the macroscopic response occurs earlier (or at a lower stress state) due to the significant tensile stresses induced by the thermal cooling process prior to the mechanical loading.

**Figure 6:**
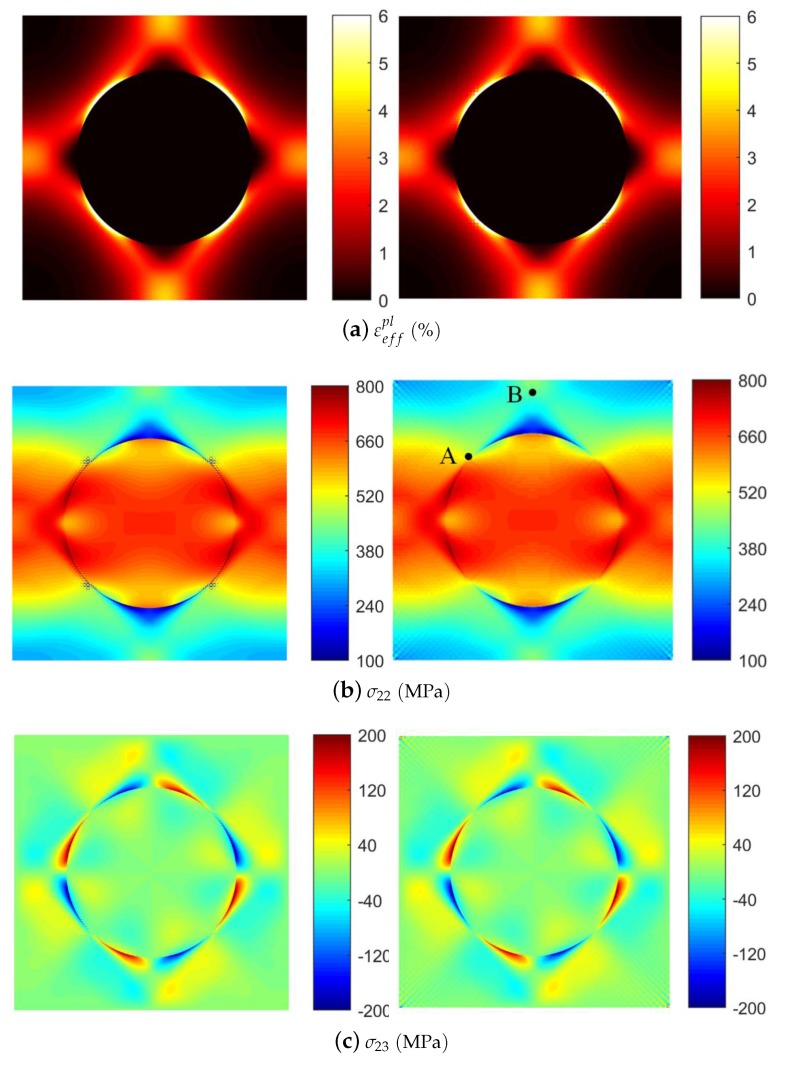
Local field distributions (μ=0.15) after $\Delta T=-550{\;}^{\circ }C$ cooldown followed by the applied strain ${\bar{\varepsilon }}_{22}=1\%$: FEM (left); FVDAM (right).

The local field distributions considering thermal effects for μ=0.15 at the applied strain of ${\bar{\varepsilon }}_{22}=1\%$ are shown in Figure 6. The results of FVDAM correlates well with the FEM prediction and no apparent difference is observed.

The FEM’s prediction, however, shows small discontinuities in the transverse normal stress field in the localized region near the fiber/matrix interface (see Figure 6b), which is not observed in the FVDAM results. This is because the large modulus contrast produces the large stress concentration and deformation gradient at the fiber/matrix interface. The FEM analysis requires a more detailed mesh discretization in the affected region to avoid stress discontinuity. The FVDAM theory, on the contrary, satisfies the equilibrium equations in each subvolume at large, which allows the excellent convergency in the region of large stress concentration.

**Figure 7:**
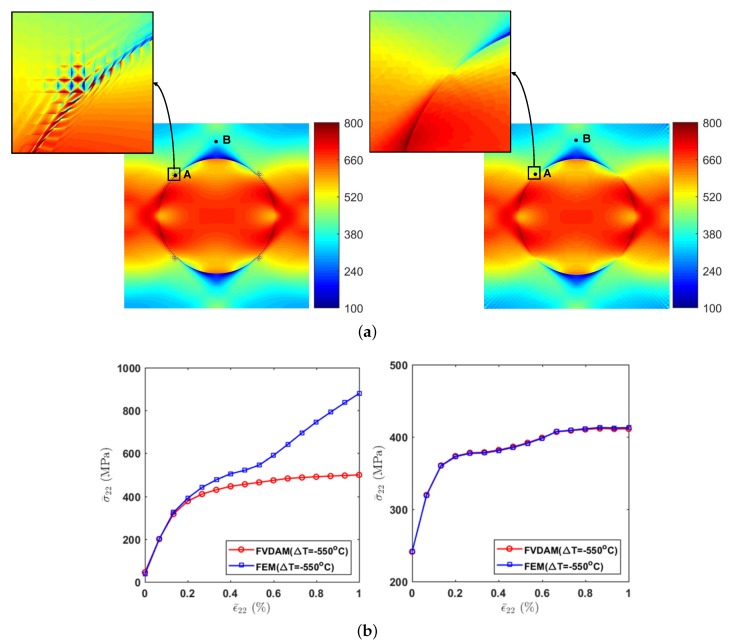
Comparison of the convergence using the finite-element method (FEM) and the finite-volume direct averaging micromechanics (FVDAM). (**a**) Comparison of the transverse thermo-mechanical tensile local field in the large stress concentration area: FEM (left); FVDAM (right); (**b**) Comparison of the transverse thermo-mechanical tensile response of the composite at point A (left) and point B (right).

To make the small discontinuities in Figure 6b more visible, we took the transverse normal stress $\sigma_{22}$ field and enlarged the region of the large stress concentration area (see Figure 7a). Small discontinuities appear in the FEM’s prediction, which are not observed in the FVDAM results.

Then, In order to get a clearer picture of the problem, we take 2 points from the transverse normal stress $\sigma_{22}$ field, where point A (−0.4, 0.5) is in the region of discontinuity and point B (0, 0.9) is in a more smooth region as shown in Figure 6b (right). Then we trace the loading from ${\bar{\varepsilon }}_{22}=0$ to ${\bar{\varepsilon }}_{22}=1\%$ using the FEM and the FVDAM; the results are demonstrated in Figure 7b. As shown in the figure, the results of point B have great agreement using these two methods, which reveals the effectiveness of the proposed model. At point A, the result of the FEM is divergent while the result using the FVDAM theory keeps an excellent convergence.

It is worth noting that the FVDAM and FEM are two different methods that share certain similarities. The formulation of local/global stiffness matrices and the procedure of discretizing unit cells are similar. However, the local stiffness matrices in FVDAM satisfy the equilibrium equations in each subvolume at large. The closed form expressions between tractions and displacements are readily available, hence the continuity conditions of both displacements and tractions are applied directly. On the contrary, the FEM method is based on the minimization of global potential and satisfies the continuity conditions of the node displacements solely.

### 4.2. Cyclic Loading

**Figure 8:**
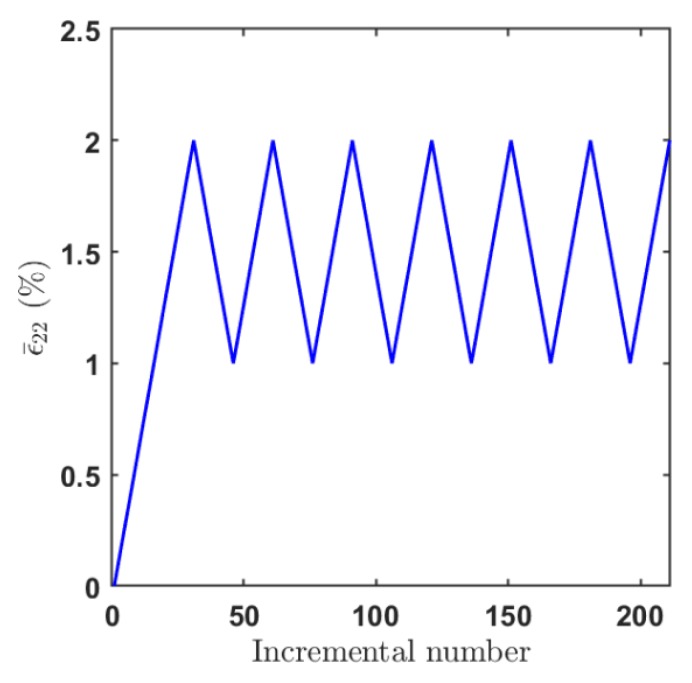
Cyclic loading history.

We further investigated the cyclic behavior of the unidirectional composite materials under unidirectional strain loading. Figure 8 shows the applied transverse strain ${\bar{\varepsilon }}_{22}$ which varies between 1% and 2% as a function of the incremental number. To demonstrate the thermal effect, the temperature changes of $\Delta T\;=\;-550{\;}^{\circ }C$ and $\Delta T\;=\;0{\;}^{\circ }C$ were applied to the composite material system prior to the mechanical loading.

**Figure 9:**
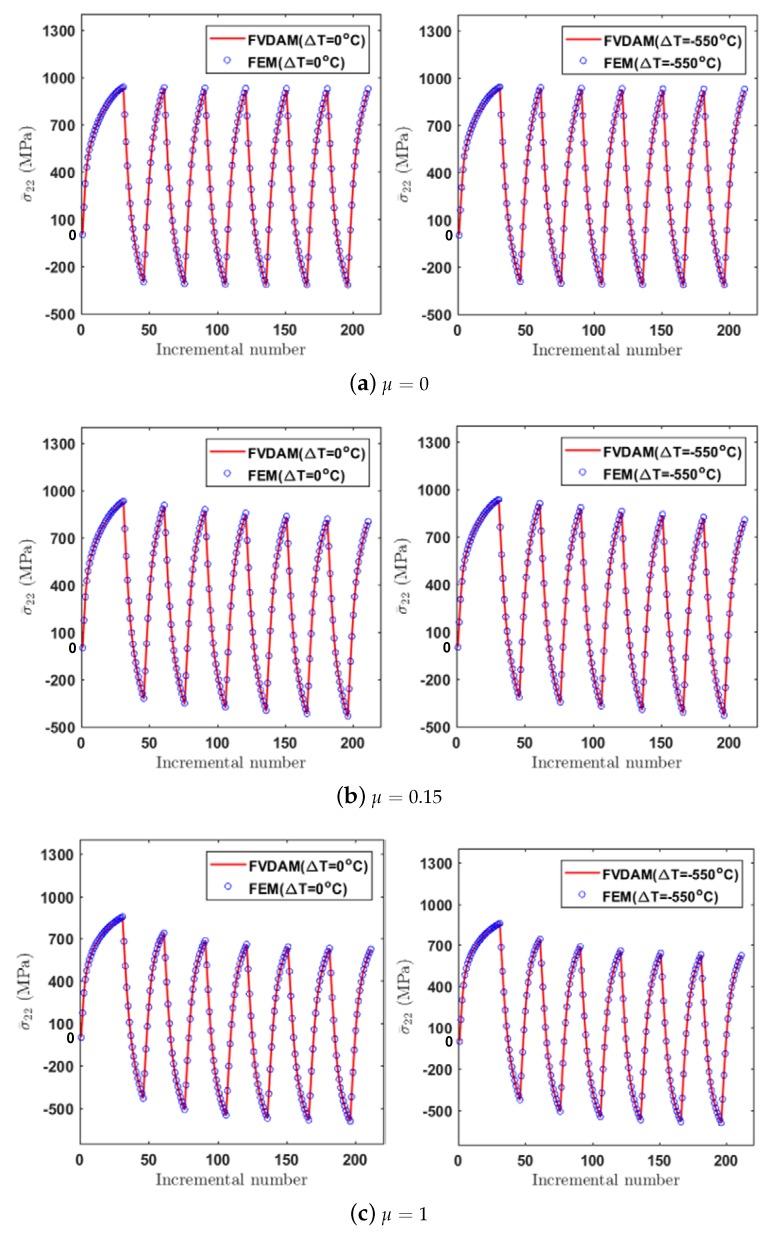
Comparison of the cyclic response of the unidirectional composite materials generated by FEM and FVDAM.

Figure 9 illustrates the validity of the FVDAM for predicting the cyclic response of the unidirectional composite materials. As shown in the figure, the FVDAM predictions are in good agreement with the FEM results. The ratcheting effect relies heavily on the plastic parameter μ. In the case of μ=0 where the constitutive model degrades to the first version of the Ohno–Wang model, the ratcheting behavior is negligible. The maximum and the minimum stresses ${\bar{\sigma }}_{22}$ at the ${\bar{\varepsilon }}_{22}=1\%$ and ${\bar{\varepsilon }}_{22}=2\%$ strain in the cyclic curve stay on two horizontally parallel lines, indicating no ratcheting effect in this case. In the case of μ=0.15, the ratcheting behavior becomes obvious. The peak and trough stresses decrease gradually with the increase of the cyclic number. Increasing the plastic parameter will further accentuate the differences in the composite transverse response due to the ratcheting effect. In the case of μ=1 where the constitutive model degrades to the AF model, the ratcheting effect becomes the most significant.

The influence of the thermal residual stresses on the cyclic response of the present material system is negligible under the transverse loading case. This is consistent with expectations because a similar response was found in [49] and Pindera and Bansal [50] for the transverse response of a unidirectional metal matrix composite. The effect of residual stresses, which is pronounced under the off-axis loading, will be discussed in the next section.

### 4.3. Off-Axis Loading

**Figure 10:**
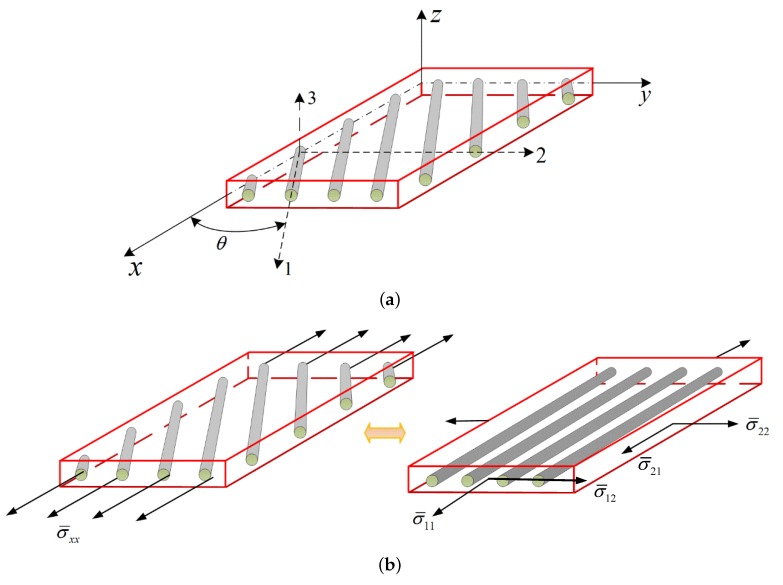
The relation between the principal coordinates and global coordinates.

We end this paper by illustrating the cyclic response under off-axis loading. In order to establish the stress–strain relations in the global coordinate systems, we rotate the principal coordinates (1,2,3) around the ‘3’ axis by θ degrees. As shown in Figure 10a, axis ‘1’ is parallel to the fiber, axis ‘2’ is perpendicular to the fiber in-plane direction and axis ‘3’ is perpendicular to the fiber out-of-plane direction. According to the coordinate transformation relations in the theory of elasticity, the stresses ${\bar{\sigma }}_{\theta }={\left[{\bar{\sigma }}_{xx},{\bar{\sigma }}_{yy},{\bar{\sigma }}_{zz},{\bar{\sigma }}_{yz},{\bar{\sigma }}_{xz},{\bar{\sigma }}_{xy}\right]}^{T}$ in the global coordinates (x,y,z) are related to the stresses $\bar{\sigma }={\left[{\bar{\sigma }}_{11},{\bar{\sigma }}_{22},{\bar{\sigma }}_{33},{\bar{\sigma }}_{23},{\bar{\sigma }}_{13},{\bar{\sigma }}_{12}\right]}^{T}$ in the principal coordinates (1,2,3) (see Figure 10b) through:

(29) $$\left[\begin{array}{c} {\bar{\sigma }}_{11}\\ {\bar{\sigma }}_{22}\\ {\bar{\sigma }}_{33}\\ {\bar{\sigma }}_{23}\\ {\bar{\sigma }}_{13}\\ {\bar{\sigma }}_{12} \end{array}\right]=\left[\begin{array}{cccccc} \mathrm{co}s^{2}\theta & \mathrm{si}n^{2}\theta & 0 & 0 & 0 & \sin2\theta \\ \mathrm{si}n^{2}\theta & \mathrm{co}s^{2}\theta & 0 & 0 & 0 & -\sin2\theta \\ 0 & 0 & 1 & 0 & 0 & 0\\ 0 & 0 & 0 & \cos\theta & -\sin\theta & 0\\ 0 & 0 & 0 & \sin\theta & \cos\theta & 0\\ -\frac{\sin2\theta }{2} & \frac{\sin2\theta }{2} & 0 & 0 & 0 & \cos2\theta \end{array}\right]\left[\begin{array}{c} {\bar{\sigma }}_{xx}\\ {\bar{\sigma }}_{yy}\\ {\bar{\sigma }}_{zz}\\ \begin{array}{c} {\bar{\sigma }}_{yz}\\ {\bar{\sigma }}_{xz}\\ {\bar{\sigma }}_{xy} \end{array} \end{array}\right]=T_{1}\left[\begin{array}{c} {\bar{\sigma }}_{xx}\\ {\bar{\sigma }}_{yy}\\ {\bar{\sigma }}_{zz}\\ \begin{array}{c} {\bar{\sigma }}_{yz}\\ {\bar{\sigma }}_{xz}\\ {\bar{\sigma }}_{xy} \end{array} \end{array}\right]$$

Similarly, the strain ${\bar{\varepsilon }}_{\theta }={\left[{\bar{\varepsilon }}_{xx},{\bar{\varepsilon }}_{yy},{\bar{\varepsilon }}_{zz},2{\bar{\varepsilon }}_{yz},2{\bar{\varepsilon }}_{xz},2{\bar{\varepsilon }}_{xy}\right]}^{T}$ in the global coordinates (x,y,z) can be transformed to stress $\bar{\varepsilon }={\left[{\bar{\varepsilon }}_{11},{\bar{\varepsilon }}_{22},{\bar{\varepsilon }}_{33},2{\bar{\varepsilon }}_{23},2{\bar{\varepsilon }}_{13},2{\bar{\varepsilon }}_{12}\right]}^{T}$ in the principal coordinates (1,2,3):

(30) $$\left[\begin{array}{c} {\bar{\varepsilon }}_{11}\\ {\bar{\varepsilon }}_{22}\\ {\bar{\varepsilon }}_{33}\\ 2{\bar{\varepsilon }}_{23}\\ 2{\bar{\varepsilon }}_{13}\\ 2{\bar{\varepsilon }}_{12} \end{array}\right]=\left[\begin{array}{cccccc} \mathrm{co}s^{2}\theta & \mathrm{si}n^{2}\theta & 0 & 0 & 0 & \frac{\sin2\theta }{2}\\ \mathrm{si}n^{2}\theta & \mathrm{co}s^{2}\theta & 0 & 0 & 0 & -\frac{\sin2\theta }{2}\\ 0 & 0 & 1 & 0 & 0 & 0\\ 0 & 0 & 0 & \cos\theta & -\sin\theta & 0\\ 0 & 0 & 0 & \sin\theta & \cos\theta & 0\\ -\sin2\theta & \sin2\theta & 0 & 0 & 0 & \cos2\theta \end{array}\right]\left[\begin{array}{c} {\bar{\varepsilon }}_{xx}\\ {\bar{\varepsilon }}_{yy}\\ {\bar{\varepsilon }}_{zz}\\ 2{\bar{\varepsilon }}_{yz}\\ 2{\bar{\varepsilon }}_{xz}\\ 2{\bar{\varepsilon }}_{xy} \end{array}\right]=T_{2}\left[\begin{array}{c} {\bar{\varepsilon }}_{xx}\\ {\bar{\varepsilon }}_{yy}\\ {\bar{\varepsilon }}_{zz}\\ 2{\bar{\varepsilon }}_{yz}\\ 2{\bar{\varepsilon }}_{xz}\\ 2{\bar{\varepsilon }}_{xy} \end{array}\right]$$

Therefore, the stress–strain relation in the global coordinates can be written as:

(31) $${\bar{\sigma }}_{\theta }={\bar{C}}^{*}{\bar{\varepsilon }}_{\theta }-{\left({\bar{\sigma }}^{pl}+{\bar{\sigma }}^{th}\right)}_{\theta }\;$$

where ${\bar{C}}^{*}=T_{1}^{-1}C^{*}T_{2}$ is the stiffness matrix in the global coordinates and ${\left({\bar{\sigma }}^{pl}+{\bar{\sigma }}^{th}\right)}_{\theta }=T_{1}^{-1}\left({\bar{\sigma }}^{pl}+{\bar{\sigma }}^{th}\right)$ are the thermo-plastic stresses in the global coordinates. Once the applied strain ${\bar{\varepsilon }}_{\theta }$ is obtained, the strain components $\bar{\varepsilon }$ in the principal coordinate system can be calculated using $\bar{\varepsilon }=T_{2}{\bar{\varepsilon }}_{\theta }$. The stress–strain relations in the global coordinates can be determined after solving Equations (13), (16) and (31).

**Figure 11:**
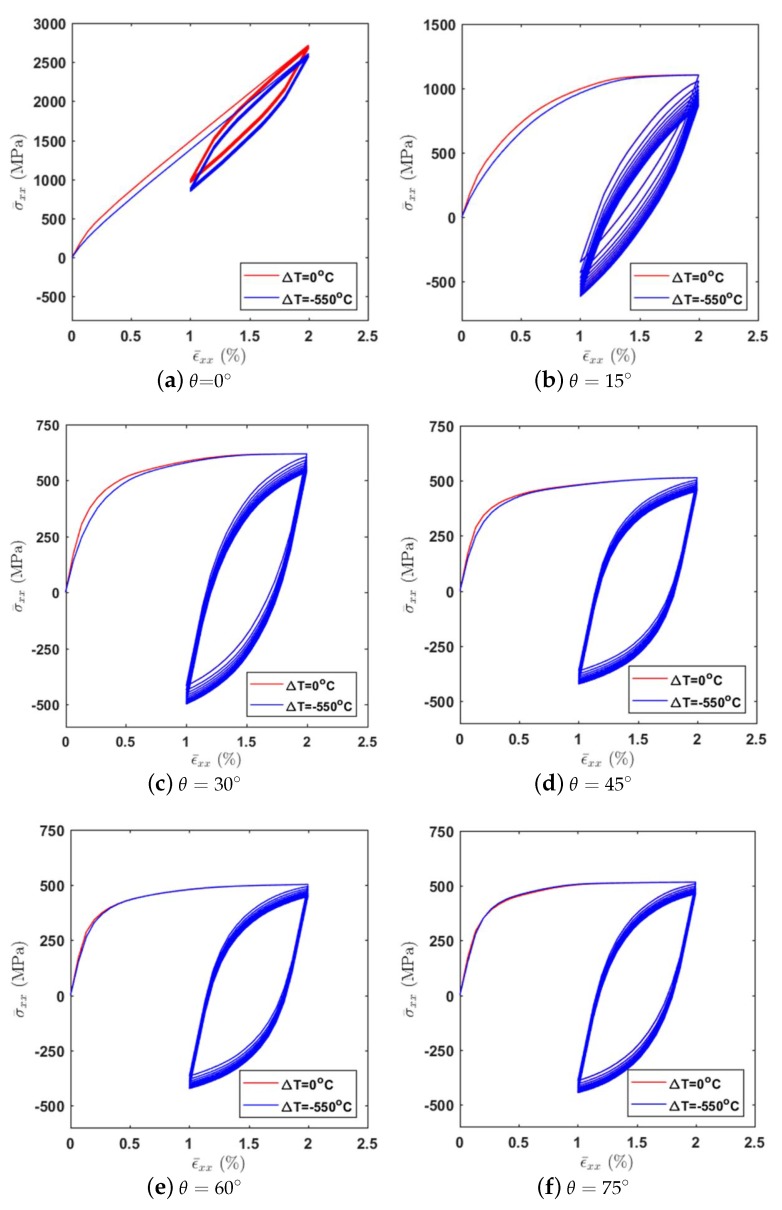
The cyclic stress–strain response with consideration of the thermal effect for the composite materials with different fiber orientations.

The cyclic stress–strain response of the composite materials with different off-axis angles ($\theta \;=\;0^{\circ }$, $15^{\circ }$, $30^{\circ }$, $45^{\circ }$, $60^{\circ }$ and $75^{\circ }$) are illustrated in Figure 11. It is revealed that the cyclic response of the unidirectional composites is sensitive to the off-axis angle. The composite response is the stiffest in the case of $0^{\circ }$ since the loading direction is along the fiber direction. With the increase of the off-axis angle, the overall stresses in the global coordinate system decrease first then increase. The thermal residual stresses have a more pronounced effect under the small off-axis loading angle cases but to a lesser extent under the moderate and large off-axis loading angle cases. The ratcheting effects are more obvious under the off-axis loading cases due to the significant out-of-plane shear-coupling.

## 5. Discussions

The existing micromechanics models for simulating the elastic-plastic response of the metal matrix composite materials are often under the framework of the Eshelby and the Mori-Tanaka theory. Most of these approaches, however, are not capable of simulating the distributions and evolutions of locals in the composites. Conventionally, the finite-element micromechanics model is the prevailing way and a golden standard for simulating both local fields and homogenized stress–strain responses of composite materials. Arbitrary microstructures can be easily addressed by the finite-element technique through the readily available commercial packages. The cyclic behavior, as well as other physical effects, can be simulated using the user programmable feature. A major shortcoming of the finite element theory is that the construction of local/global system of equations is under the framework of the variational principle. Hence, the simulation of composite materials often requires a detailed mesh near the interface due to the large modulus contrast between constituent materials. Compared with the FEM, the FVDAM is a semi-analytical method which provides an accuracy comparable to the FEM but with better efficiency. What is more, for the fatigue life prediction of the composite materials subjected to cyclic loading, since the proposed model can successfully calculate the local fields of the composites, the simulation of damage evolution and ultimately failure could be a worthwhile pursuit.

## 6. Summaries and Conclusions

The FVDAM model is extended by incorporating the cyclic plasticity model with the nonlinear kinematic hardening rule originally proposed by Abdel-Karim and Ohno, which enables efficient and accurate simulation of elastoplastic ratcheting behavior and local field distributions of the metal matrix composite materials. Comparisons of the results generated by the FVDAM and the finite-element method not only demonstrate the accuracy of the FVDAM theory but the effectiveness and efficiency. The generated results also indicate that the FVDAM has better convergence and stability than the FEM theory since the FVDAM eliminates the stress discontinuities in the vicinity of the interface which are observed in the FE simulations.

The FVDAM is used to investigate the influence of the thermal residual stress and fiber-orientations on the ratcheting behavior of metal matrix composites. The numerical simulations show that the thermal residual stresses provide a slight enhancement in homogenized stresses. However, the influence of the thermal residual stresses on the cyclic response of the present material system is negligible under the transverse loading case. Ratcheting effects are more obvious under the off-axis loading cases due to the significant out-of-plane shear-coupling. Last but not least, the thermal residual stresses have a more pronounced effect under the lower off-axis loading cases.

## Figures and Tables

**Table 1:** Material and thermal properties for fiber and matrix.

	Matrix	Fiber
E/GPa	215	400
ν	0.33	0.25
$\alpha /10^{-6}{\;}^{\circ }C^{-1}$	8	4.86
Y/MPa	220	*∞* (elastic)

**Table 2:** Kinematic hardening parameters.

$\zeta^{\left(1\right)}=2000$	$\zeta^{\left(2\right)}=500$	$\zeta^{\left(3\right)}=200$	$\zeta^{\left(4\right)}=50$
$r^{\left(1\right)}=50$	$r^{\left(2\right)}=50$	$r^{\left(3\right)}=50$	$r^{\left(4\right)}=100$
μ=0,0.15,1			

## Abbreviations

The following abbreviations are used in this manuscript:

FVDAM	Finite-volume direct averaging micromechanics
FE	Finite element
FEM	Finite element method
